# Differences in Cognitive Health and Brain Activity According to Mild Cognitive Impairment and Physical Activity Levels in Older Women

**DOI:** 10.3390/brainsci15111181

**Published:** 2025-10-31

**Authors:** Jidong Tian, Wookwang Cheon

**Affiliations:** 1 College of Physical Education, Weinan Normal University, Weinan 714099, China; tianjidong@wnu.edu.cn; 2 College of Kinesiology, Keimyung University, Daegu 42403, Republic of Korea

**Keywords:** old women, mild cognitive impairment (MCI), physical activity level, cognitive health, brain activity

## Abstract

Background: The purpose of this study was to investigate differences in cognitive well-being and brain activity between older women with and without mild cognitive impairment (MCI) across varying levels of physical activity. Method: A total of 126 female participants aged over 65 years were recruited and categorized into MCI and non-MCI groups. Cognitive health was evaluated using the Korean versions of the Mini-Mental State Examination (MMSE-K) and the Montreal Cognitive Assessment (MoCA-K), while physical activity levels were quantified with the Physical Activity Scale for the Elderly (PASE-K). Brain activity was assessed through electroencephalography (EEG). Data were analyzed using two-way ANOVA. Results: Results indicated that non-MCI participants consistently demonstrated higher cognitive scores across all physical activity levels. Moreover, individuals with higher physical activity exhibited greater theta wave activity compared with those in the MCI group. Significant group differences were also observed in concentration and stress levels. Conclusion: These findings suggest that higher physical activity levels are associated with better cognitive performance and brain activity in older women. Promoting physical activity may therefore contribute to strategies aimed at supporting healthy cognitive aging, although longitudinal research is required to establish causal relationships.

## 1. Introduction

The population of South Korea is rapidly aging, and the incidence of age-related conditions, such as dementia and mild cognitive impairment (MCI), is on the rise. As of 2021, approximately 10.33% of the population aged 65 years and older is expected to receive dementia care. A growing body of research indicates that by 2050, the number of individuals living with dementia will exceed three million, with more than 15% of the elderly population likely to be affected, placing a significant burden on healthcare systems and costs. From 2015 to 2019, the number of individuals diagnosed with dementia increased by approximately 588,000 and the economic burden of dementia increased by a factor of 1.5, with indirect costs borne by family members and caregivers accounting for 48% of the total cost. In particular, the prevalence of dementia increases with age, and hospital utilization is also rising [1,2].

Mild cognitive impairment (MCI) is an intermediate stage between dementia and normal cognitive function in the elderly. It is characterized by impairment of various cognitive functions, including memory, without significant impairment in daily life. MCI is recognized as a significant health problem with a high risk of progressing to dementia [3]. Approximately 10–15% of patients with mild cognitive impairment (MCI) progress to dementia. As of 2019, the number of patients with MCI was approximately 270,000, representing a 19-fold increase over the past 10 years [4]. Women are 2.2 times more likely to be diagnosed than men. This may be attributed to the impact of hormonal changes associated with menopause on cognitive function, which can increase the risk of cognitive diseases such as Alzheimer’s disease. Consequently, MCI management in older women is a significant public health concern. The conversion rate of MCI to Alzheimer’s disease is 3–15%, in comparison to 1–2% in the general population. This necessitates intensive research and management strategies [5].

A recent study demonstrated that a combined approach of cognitive training and physical activity is effective for the management of mild cognitive impairment (MCI) in patients with Parkinson’s disease. To further investigate this, a study using rs-fMRI was conducted to analyze the brain functional activity in patients with amnestic MCI [6]. Furthermore, alterations in functional network stability and dynamic connectivity of alpha and gamma were observed in patients with early Parkinson’s patients, and physical activity in elderly Colombian individuals was found to have a positive effect on MCI prevention [7]. These studies underscore the pivotal role of brain and physical activity in the onset and progression of MCI, particularly in the context of demographic variations. Elevated theta waves in the frontal lobe may serve as an initial indicator of Alzheimer’s disease (AD). Additionally, augmented spectral power and coherence in the slow frequency band of the resting-state electroencephalogram (EEG) may prove beneficial as a biomarker for detecting cognitive decline in mild cognitive impairment (MCI) and AD [8,9].

The health benefits of physical activity are well documented, with evidence of a number of outcomes. These include cognitive and physical decline, functional mobility and well-being, glycemic control, and depressive symptoms [10]. It has been estimated that 292,600 new cases of dementia can be averted globally if all individuals engage in regular physical activity [11]. Cognitive function represents a primary domain affected by dementia, with physical activity linked to either cognitive enhancement or decline [12]. Furthermore, evidence indicates that aerobic training (AT) has a beneficial effect on hippocampal volume in healthy older adults [13]. The results demonstrated that AT performed three times per week led to a significant increase in left, right, and total hippocampal volumes in older adults with MCI when compared to a control group that performed balance and exercise [14]. In a study examining the effects of high-intensity aerobic training (AT) on cognitive function in older adults with mild cognitive impairment (MCI), subjects were randomly assigned to either a high-intensity AT group (45–60 min per session, heart rate 75–85%) or a stretching and control group, with both groups completing four sessions per week for a six-month period. The high-intensity AT group showed improved cognitive function [15].

Recent studies have indicated that various physical activities and exercise programs can have a beneficial impact on the physical and cognitive functioning of patients with mild cognitive impairment (MCI). These programs have the potential to enhance the quality of life of older adults and contribute to the prevention or delay of MCI onset. Although these studies underscore the significance of physical activity in averting MCI and dementia, there is a paucity of research examining the precise impact of physical activity levels on cognitive function in patients with MCI. Consequently, further investigation is imperative to ascertain the precise effects of physical activity on cognitive function to enhance the management and prevention of MCI.

The purpose of this study was to investigate differences in cognitive well-being and brain activity between individuals with and without mild cognitive impairment (MCI) across varying levels of physical activity, and to clarify key factors driving the onset and progression of MCI to inform tailored prevention and treatment strategies.

## 2. Methods

### 2.1. Participants

In this study, the G-Power 3.1.9.7 program was used to calculate the requisite number of subjects based on the specified effect size (0.15), alpha error probability (α err prob: 0.05), and power (0.85). In total, 115 subjects were required; however, 126 older adult individuals aged 65 to 89 years residing in City D were ultimately selected, taking into account the invalid questionnaire dropout rate. The participants were divided into two groups: 63 individuals with normal cognitive function and 63 individuals with mild cognitive impairment. They were recruited from homes and facilities for the older adult. Individuals who had undergone major trauma or surgery in the last six months were excluded from the study. Participants ranged in age from 65 to 89 years. Exclusion criteria included blindness, aphasia, severe hearing impairment, severe neurological or psychiatric disorders, and perceptual impairments that would prevent completion of cognitive assessments. Voluntary consent was obtained after the purpose and procedures of the study were fully explained. The final participants underwent brain activity measurement, physical activity survey, and cognitive function assessment. Only female participants were recruited to minimize gender-related variability in EEG activity and to ensure group homogeneity. This decision was made based on prior evidence of sex differences in cognitive aging. The study characteristics are summarized in Table 1.

### 2.2. Research Procedures

**Figure 1:**
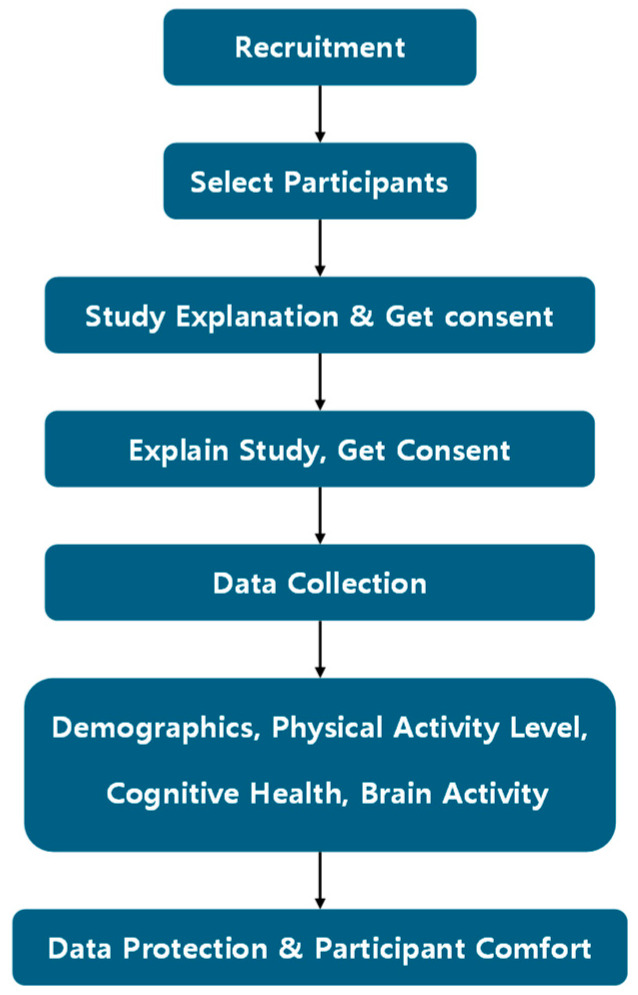
Research Flowchart.

The objective of this study was to analyze the effects of physical and brain activity on cognitive health in older female individuals with mild cognitive impairment, as illustrated in Figure 1.

The study was conducted on a cohort of older female individuals (aged 65 years or older) residing in D City, who had no history of major trauma or surgery within the previous six months. Participants were recruited through the cooperation of local welfare facilities and senior physical education facilities. Written informed consent was obtained for voluntary participation after the purpose and procedures of the study had been explained. Data collection included the administration of a questionnaire to ascertain demographic characteristics (age, education level, and living arrangements), the Montreal Cognitive Assessment (MoCA), and the Mini-Mental State Examination (MMSE) to evaluate cognitive function; the Geriatric Physical Activity Questionnaire to assess physical activity; and an electroencephalograph (EEG) to record brain activity. Following the removal of incomplete responses and data cleaning, the impact of physical activity and brain activity on cognitive health was assessed using a two-way ANOVA.

### 2.3. Methods

#### 2.3.1. Mild Cognitive Impairment Assessment

The MoCA-K is based on the Montreal Cognitive Assessment (MoCA) developed by Nasreddine et al. to screen for mild cognitive impairment. The tool was translated into Korean, underwent a series of revisions, was supplemented with additional items, and underwent a final validation process before being finalized. The MoCA-K assesses seven cognitive domains, each comprising 30 questions. Participants with six or fewer years of education were assigned one additional point to account for potential cognitive differences based on educational attainment. In this study, subjects with an MoCA-K score of 22 or less were classified as exhibiting mild cognitive impairment. The original MoCA tool demonstrated high reliability, as evidenced by a Cronbach’s α of 0.83 [16]. The Korean version of the MoCA-K exhibited comparable reliability, with Cronbach’s α values ranging from 0.81–0.84 [17].

#### 2.3.2. Physical Activity Scale for the Elderly-Korea (PASE-K)

To assess the physical activity level of the older adult, we used the Korean version of the Physical Activity Scale for the Elderly (PASE), originally developed by Washburn et al. for older adults, which was subsequently translated and modified by Choe et al. The level of physical activity was calculated according to the PASE scoring method as outlined in the tool developed by Washburn et al. The contribution of physical activity to the total score was calculated as the product of activity frequency and PASE weight. The potential scores range from 0 to 360, with higher scores indicating greater physical activity [18,19].

#### 2.3.3. Mini-Mental State Examination-Korea (MMSE-K)

The Mini-Mental State Examination (MMSE), initially developed by Folstein et al. in 1975, was subsequently modified and adapted by Lee et al. The 1989 MMSE-K consists of seven domains: orientation to time and place, memory, recall, calculation and concentration, understanding and judgment, and language. A total score of 30 was used, and a decrease of three or more points in the total score from the previous year’s score was as long-term cognitive decline [20,21,22,23]. In this study, the MoCA-K cut-off of ≤22 was adopted, consistent with previous Korean validation studies among older adult populations. This threshold has been shown to effectively distinguish individuals with MCI in this demographic. Although long-term decline is often defined as a decrease of three or more points from the previous year’s score, this study applied MMSE-K cross-sectionally only, and no longitudinal comparisons were made.

#### 2.3.4. Brain Activity Test

The Brain Test is a tool that enables the measurement of brain activity. It assesses spatial cognition and memory among other cognitive functions. The test comprises a series of sub-items, each of which is analyzed in terms of its contribution to overall cognitive performance [24]. In the memory task, participants were presented with a complex figure composed of squares arranged in grids ranging from 3 × 3 to 5 × 5. The figure was displayed for approximately three seconds and then disappeared. Subsequently, another figure of the same format was presented, and participants were asked to judge whether the second figure was identical to the previously shown figure. In the spatial cognition task, participants were shown two complex figures, also composed of squares arranged in 3 × 3 to 5 × 5 grids. The task required them to determine whether the figure presented on the right side corresponded to the left figure after a 90-degree counterclockwise rotation. Both tasks were integrated into the standardized test protocol of the EEG device and were designed to assess cognitive load, memory processing, spatial reasoning, and reaction time. The device automatically recorded indices such as cognitive intensity and reaction time, which were then used in the subsequent analysis. EEG data were including band-pass filtering at 0.5–50 Hz, artifact removal (eye blinks and muscle movements), and baseline normalization. Calibration was performed prior to each session to ensure reliability. These sub-items included measures of cognitive strength, cognitive speed, concentration, brain stress, and left and right brain activity. Concentration was assessed through sustained attention tasks, while stress/mental workload was measured during tasks involving dual cognitive demands. Calibration was performed before each session. The neurophysiological EEG indicators for each item are listed in Table 2.

### 2.4. Statistical Analysis

The mean and standard deviation of all items were calculated using SPSS 27.0 Win statistical program, and a frequency analysis was conducted to determine the general characteristics of the study subjects. An independent sample t-test was employed to ascertain discrepancies in demographic characteristics contingent on the presence or absence of mild cognitive impairment. Additionally, a Two-way ANOVA was used to investigate the disparities in each item with regard to the presence or absence of mild cognitive impairment and physical activity level. Two-way ANOVA was performed to test the main and interaction effects of MCI status (MCI vs non-MCI) and physical activity levels (low, moderate, high). All statistical significance levels were set at α = 0.05.

## 3. Results

### 3.1. Differences in Demographics with and Without MCI

Differences in demographic characteristics based on the presence or absence of MCI are presented in Table 3. Participants in the MCI group were significantly older and more likely to be widowed compared to participants without MCI. Conversely, education level, living arrangement, and cost of living were significantly (*p* < 0.05) higher in the normal older group than in the MCI older group.

### 3.2. Differences in Cognitive Health Based on MCI Status and Physical Activity Levels

**Figure 2:**
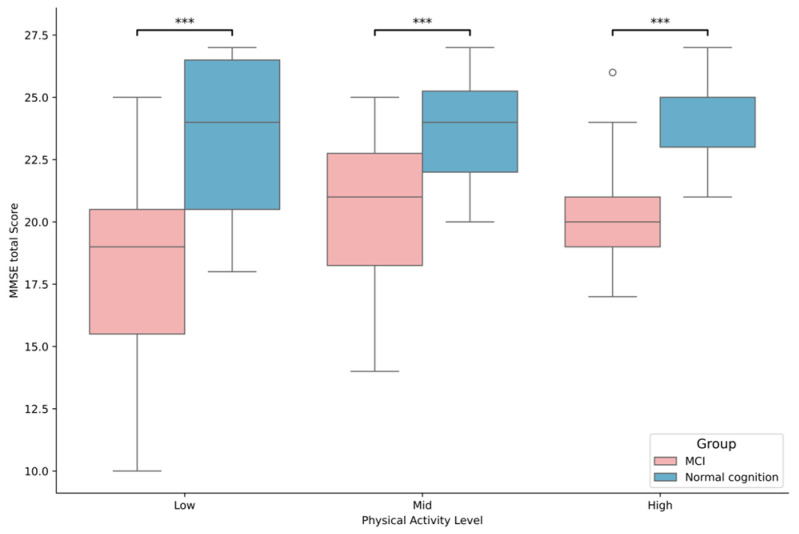
Differences in Cognitive Health Based on MCI Status and Physical Activity Levels, *** *p* < 0.001.

The results of the analysis of the discrepancy in MMSE score between the MCI and physical activity levels are illustrated in Figure 2. The primary effect of group was statistically significant [F(1, 125) = 53.752, *p* < 0.001], yet the interaction with physical activity level was not significant. Subsequent post hoc tests of the primary effect of group demonstrated that the MMSE score of normal older adult was significantly (*p* < 0.05) better than that of MCI older adult across all physical activity levels.

### 3.3. Differences in Brain Waves Based on MCI Status and Physical Activity Levels

**Figure 3:**
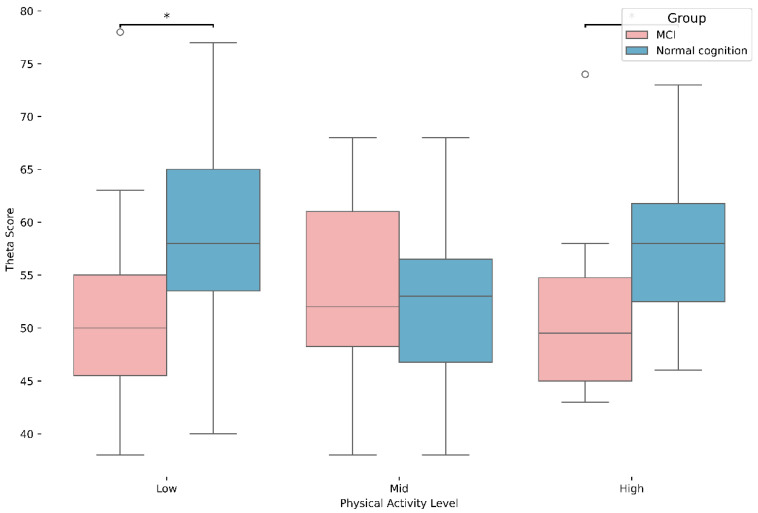
Differences in Theta wave based on MCI status and physical activity levels, * *p* < 0.05.

The results of the analysis of the discrepancy in brain waves between MCI and physical activity levels are illustrated in Table 4 and Figure 3. The primary effect of group was statistically significant in the theta waveform (F(1, 125) = 7.081, *p* < 0.05), yet no notable differences were observed in the remaining waveforms. Post hoc tests showed that the non-MCI high physical activity group exhibited significantly greater theta activity compared to the MCI group across Low and High activity levels (*p* < 0.05). However, the high physical activity group exhibited a statistically significant (*p* < 0.05) elevation compared to the MCI group.

### 3.4. Differences in Brain Activities Based on MCI Status and Physical Activity Levels

**Figure 4:**
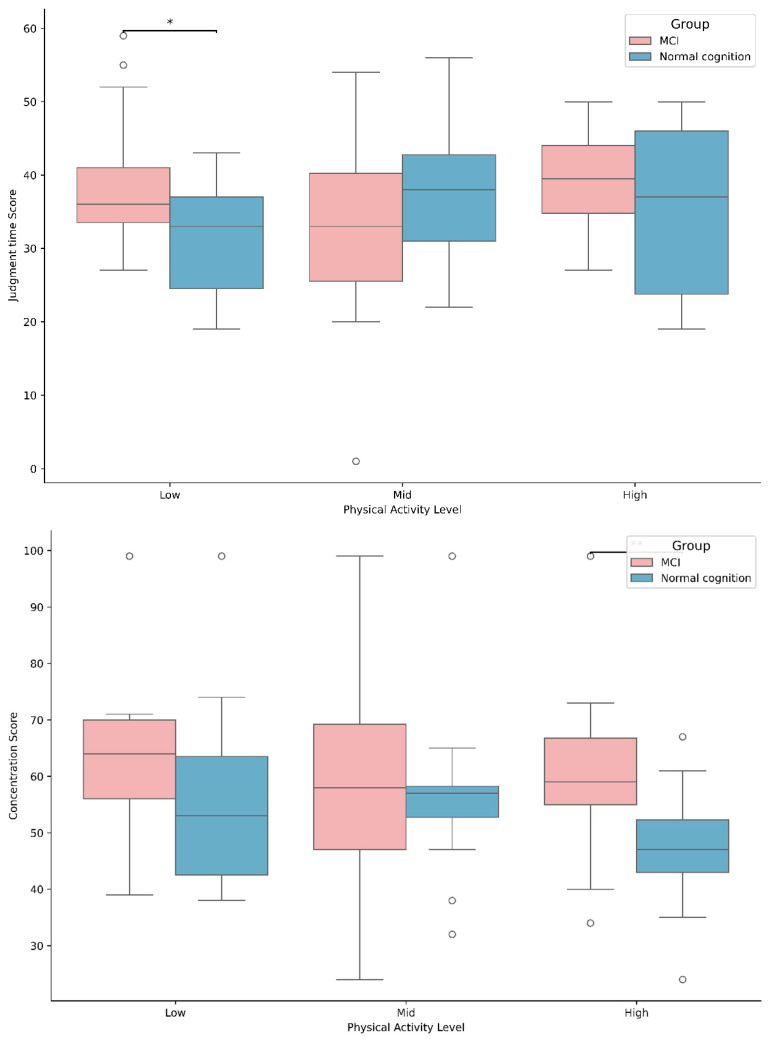
Differences in judgement time and concentration based on MCI status and physical activity levels, * *p* < 0.05, ** *p* < 0.01.

The results of the analysis of the discrepancy in brain activity between MCI and physical activity levels are illustrated in Table 5 and Figure 4. A significant difference was found in cognitive speed [*F*(2, 125) = 3.515, *p* < 0.05] for the interaction between group and level. Post hoc tests of the interaction showed that MCI was significantly lower in the low physical activity group than in the normal older adult (*p* < 0.05), but no significant differences were found for the other physical activity levels, and no differences in physical activity level were found between the group. The main effect of group was significantly different for concentration [*F*(1, 125) = 9.872, *p* < 0.01] and stress [*F*(1, 125) = 5.579, *p* < 0.05], and the main effect of level was significantly different for L brain activity [*F*(2, 125) = 3.880, *p* < 0.05], The main effect of group concentration showed a significant difference in L brain activity [*F*(2, 125) = 3.880, *p* < 0.05], but there was no significant difference in all other activity levels. The main effect of group stress showed no significant difference between the high and moderate physical activity groups, but the MCI was significantly (*p* < 0.05) lower than the MCI in the high physical activity group. The main effect of level L brain activity was significantly (*p* < 0.05) lower in the high physical activity group than in the low physical activity group.

## 4. Discussions

The following section presents the findings of the analysis of the differences in demographic variables, cognitive health, and brain activity in relation to the presence of mild cognitive impairment and physical activity levels among older women.

Aging, marital status, education, and household income, categorized according to national quartiles, are significant factors that affect cognitive function in older adults with mild cognitive impairment (MCI). First, aging plays an important role in accelerating cognitive decline caused by structural and physiological changes in the brain. Specifically, the loss of gray and white matter in the brain increases with age, while the number of nerve cells and synaptic density decrease. These changes can negatively impact a variety of cognitive abilities, including memory, attention, and executive function [25]. These changes in brain structure increase the risk of mild cognitive impairment, which is a precursor to dementia. Consequently, older adults are more likely to experience cognitive decline. Furthermore, marital status exerts a significant influence on cognitive health. The emotional security and economic stability that marriage provides on a daily basis have been shown to have a positive impact on the maintenance of cognitive health in older adults [26]. A review of the literature reveals that married individuals are more likely to adhere to healthy lifestyle habits than their single counterparts are. This finding suggests that mutual support between spouses may contribute to a slower rate of cognitive decline. Nevertheless, marital status may not be the sole determining factor for cognitive function. A broader support system comprising social connections beyond marriage, including friends, family, and the broader community, may also prevent cognitive decline [27]. Specifically, our findings indicate that the marital status of the individuals in question. This underscores the importance of quality social support rather than merely the form of marriage as a crucial element in maintaining cognitive health [28]. Education level is a significant predictor of cognitive function and prevention of decline. Individuals with higher levels of education tend to possess greater cognitive reserve, which enables them to maintain cognitive function for a relatively extended period following brain injury [29]. Such individuals are more likely to be engaged in complex and challenging occupations, which continue to stimulate cognitive function and contribute to its maintenance of function [30]. Individuals with higher levels of education have greater access to health-related information, which can be used to prevent cognitive decline by undergoing regular health examinations and maintaining a healthy lifestyle. Moreover, higher education levels are associated with a higher frequency of social activities and networking, which may have a positive impact on maintaining cognitive function. This is consistent with previous research indicating that brain stimulation and emotional support contribute to the prevention of cognitive decline [31]. Economic circumstances have a considerable influence on the cognitive performance of older individuals. Economic hardship can result in diminished access to healthcare, suboptimal nutritional status, and heightened psychological stress, all of which may contribute to cognitive decline [32]. In particular, older adults with limited economic resources may encounter difficulties maintaining a nutritious diet, which can result in nutritional deficiencies and accelerate cognitive decline [33]. Conversely, a stable housing environment has been shown to prevent cognitive decline by facilitating the maintenance of emotional stability and social networks. Research indicates that it plays a pivotal role in maintaining cognitive function by reducing social isolation and stress [34]. Consequently, a multitude of factors, including aging, marital status, education level, and economic conditions, exert a significant influence on the maintenance of cognitive health and prevention of cognitive decline in the older adult. Therefore, comprehensive support that considers these factors is essential.

This study examined the disparate effects of physical activity on cognitive health, electroencephalogram (EEG) waveforms, and brain activity. The findings indicated that, at all levels of physical activity, non-MCI older adults exhibited superior cognitive health compared to older adults with MCI, thereby substantiating the beneficial impact of physical activity on cognitive health. In particular, physical activity has been demonstrated to enhance frontal lobe function and memory while also facilitating neuroplasticity through the postponement of hippocampal aging and augmentation of cortical thickness [35]. These findings have the potential to inform the development of strategies to delay or prevent cognitive decline in older adults with MCI. Moreover, recent studies have demonstrated that moderate-to-vigorous physical activity, in particular, plays a pivotal role in decelerating the progression of MCI and preserving cognitive function. Daily physical activity has been shown to exert a beneficial influence [36,37]. It has been postulated that physical activity may protect cognitive function through physiological mechanisms, including increased cerebral blood flow, improved vascular function, and reduced inflammation. Furthermore, it may contribute to overall cognitive maintenance by lowering stress hormone levels and promoting antidepressant effects [38]. It is noteworthy that positive effects of physical activity were also observed in improving the reaction time and concentration. This suggests that physical activity may help prevent the transition to MCI by increasing the cognitive reserve. Improvements in reaction time are closely related to neuroplasticity, and regular physical activity may promote neuroplasticity in the brain, thereby strengthening cognitive functions such as information processing speed [39]. Furthermore, older adults with MCI exhibited elevated stress levels compared to those without MCI, suggesting a potential interplay between early stage cognitive impairment and stress. This implies that chronic stress may negatively affect the hippocampus and contribute to cognitive decline [40]. Furthermore, research has indicated that social support can positively influence cognitive function by moderating stress responses. This highlights the necessity of stress management and social support enhancement programs for older adults with MCI [41]. Left brain activation is elevated when physical activity levels are low, which may be indicative of neurophysiological alterations associated with stress [42,43]. The left brain is primarily responsible for logical thinking and problem solving. Therefore, it is possible that increased left brain activation associated with low physical activity is related to stressors. These findings suggest that physical activity may play an important role in maintaining brain balance and supporting psychological stability. These findings highlight the importance of physical activity in maintaining overall brain health and improving quality of life in the older population. They also support the need for tailored programs that consider physical activity levels and exercise intensities.

This study has several limitations. First, physical activity was assessed through self-reported questionnaires, which may be subject to recall bias. Future studies should employ objective measures such as accelerometers. Second, the lack of body composition indicators (e.g., BMI, muscle and fat percentage) limited the interpretation of the results. Finally, as this was a cross-sectional study, causal inferences cannot be drawn. Longitudinal or interventional studies are needed to clarify the directionality of associations.

## 5. Conclusions

In summary, higher physical activity levels were associated with better cognitive health and brain activity among older women, although these associations were weaker in those with MCI. These findings highlight the importance of promoting physical activity to support healthy aging, while acknowledging the need for longitudinal research to establish causality.

## Figures and Tables

**Table 1:** Characteristics of the study subjects.

Variables		MCI (n,%)	Non MCI (n,%)
Age	65–75	14 11.22%	38 30.27%
	76–85	38 30.27%	23 18.36%
	Over 86	11 8.84%	1 1.04%
Education	No formal education/less than elementary school	13 10.32%	2 1.59%
	Elementary school	38 30.16%	28 22.22%
	Middle school and above	12 9.52%	33 26.19%
Residency	Community dwelling	35 27.78%	15 11.90%
	Assisted living facility	28 22.22%	48 38.10%
Number of people in the household	One	28 22.22%	16 12.69%
	Two	28 22.22%	40 31.75%
	Three or more	7 5.56%	7 5.56%
Marital status	Currently married	42 33.33%	53 42.06%
	Separated or widowed	21 16.67%	10 7.94%
Household income (KRW)	Less 1 million	49 38.89%	25 19.84%
	Over 1.01 million	14 11.11%	38 30.16%
Number of diseases	None	11 8.84%	13 9.76%
	One	21 16.67%	19 15.08%
	Two	13 9.76%	23 18.25%
	Over two	18 14.29%	8 7.35%

**Table 2:** Brain literacy subcategories Neurophysiological EEG metrics.

Details	Neurophysiological Brainwave Signatures
Instant memory	The mean cognitive intensity, averaged over each difficulty level, is expressed in microvolts per second (μV/S). The amplitude change (in height) of the cognitive gamma-peak.
Judgement time	The mean reaction time for each difficulty level was calculated as a mean value in seconds.
Concentration	(SMR + M-Beta)/Theta SMR = Sensory Motor Rhythm
Mental workload	SEF90% (spectral edge frequency-90%)
L/R brain activity	The relative prevalence of gamma power in the left and right cerebral hemispheres.

**Table 3:** Analysis of Differences in Demographic Characteristics Based on the Presence or Absence of MCI.

Variable	MCI	Non MCI	t-Value	95% CI	
				Lower	Upper
Age	79.43 ± 5.87	74.08 ± 5.45	5.298 ***	0.574	1.311
Education	3.00 ± 0.80	3.90 ± 1.10	−5.263 ***	−1.304	−0.568
Housing type	1.44 ± 0.50	1.76 ± 0.43	−3.819 ***	−1.039	−0.320
Household size	1.79 ± 1.00	1.89 ± 0.70	−0.619	−0.460	0.239
Marital status	2.33 ± 0.48	2.16 ± 0.37	2.305 *	0.057	0.763
Cost	87.54 ± 46.08	159.05 ± 78.01	−6.264 ***	−1.490	−0.738
Chronic disease	1.86 ± 1.84	1.43 ± 1.00	1.627	−0.062	0.640

M ± SD, * *p* < 0.05, *** *p* < 0.001.

**Table 4:** Differences in brain waves based on MCI status and physical activity levels.

Variable	GPL	MCI	Non MCI	*F*	
	Low	67.71 ± 14.22	62.36 ± 11.17	G	2.185
Gamma	Mid	64.56 ± 20.86	63.71 ± 12.93	PL	0.472
	High	64.93 ± 17.98	58.54 ± 11.87	G ∗ PL	0.385
	Low	67.61 ± 15.91	60.27 ± 11.01	G	3.103
H-Beta	Mid	62.67 ± 18.97	60.92 ± 12.42	PL	1.117
	High	61.57 ± 17.78	55.96 ± 10.42	G ∗ PL	0.362
	Low	70.52 ± 17.81	64.64 ± 14.88	G	2.310
M-Beta	Mid	63.83 ± 18.09	60.79 ± 11.69	PL	1.615
	High	63.71 ± 15.88	59.68 ± 10.40	G ∗ PL	0.083
	Low	66.29 ± 14.51	66.36 ± 13.86	G	0.661
SMR	Mid	65.33 ± 17.14	59.67 ± 10.98	PL	0.723
	High	64.21 ± 14.66	63.46 ± 11.43	G ∗ PL	0.497
	Low	57.52 ± 13.06	63.27 ± 14.76	G	0.447
Alpha	Mid	60.94 ± 16.42	57.13 ± 10.72	PL	0.118
	High	57.79 ± 12.78	60.68 ± 9.56	G ∗ PL	1.426
	Low	51.68 ± 9.32	58.55 ± 10.24 ^#^	G	7.081 **
Theta	Mid	53.28 ± 9.44	52.92 ± 7.52	PL	0.544
	High	51.21 ± 8.32	57.68 ± 7.03 ^#^	G ∗ PL	2.199

M ± SD, G: Group, PL: Physical activity Level, ^#^: significantly differed between the MCI and non-MCI groups, ** *p* < 0.01.

**Table 5:** Differences in brain activities based on MCI status and physical activity levels.

Variable	GPL	MCI	Non MCI	*F*	
	Low	48.03 ± 5.29	49.36 ± 7.57	G	0.085
Instant memory	Mid	52.11 ± 6.09	48.63 ± 5.13	PL	0.742
	High	49.79 ± 8.78	50.86 ± 7.19	G ∗ PL	1.688
	Low	37.84 ± 8.51	31.00 ± 8.41 ^#^	G	0.914
Judgement time	Mid	32.83 ± 12.12	37.67 ± 9.59	PL	0.831
	High	39.00 ± 6.96	35.64 ± 11.24	G ∗ PL	3.515 *
	Low	65.74 ± 15.19	56.09 ± 18.33	G	9.872 **
Concentration	Mid	59.83 ± 19.07	56.21 ± 11.80	PL	1.839
	High	60.71 ± 15.56	47.82 ± 9.81 ^#^	G ∗ PL	1.028
	Low	61.00 ± 12.63	54.36 ± 13.38	G	5.579 *
Mental workload	Mid	57.28 ± 17.97	54.54 ± 12.36	PL	2.742
	High	54.93 ± 17.48	46.29 ± 8.08	G ∗ PL	0.500
	Low	50.19 ± 8.88	50.09 ± 9.49	G	0.327
L brain activity	Mid	48.39 ± 2.68	48.75 ± 6.19	PL	3.880 *
	High	45.14 ± 3.72	46.93 ± 2.93	G ∗ PL	0.223
	Low	53.45 ± 3.92	52.18 ± 5.69	G	2.773
R brain activity	Mid	52.67 ± 3.16	51.67 ± 6.51	PL	1.796
	High	55.00 ± 3.59	53.07 ± 2.93	G ∗ PL	0.113

M ± SD, G: Group, PL: Physical activity Level, ^#^: significantly differed between the MCI and non-MCI groups, * *p* < 0.05, ** *p* < 0.01.

## Data Availability

The data that support the findings of this study are available from the corresponding author upon reasonable request. The data are not publicly available due to privacy concerns.
